# Human osteochondritis dissecans fragment-derived chondrocyte characteristics ex vivo, after monolayer expansion-induced de-differentiation, and after re-differentiation in alginate bead culture

**DOI:** 10.1186/s12891-018-2079-6

**Published:** 2018-05-24

**Authors:** Matthias Aurich, Gunther O. Hofmann, Florian Gras, Bernd Rolauffs

**Affiliations:** 1 Center for Orthopaedic and Trauma Surgery, Klinikum Mittleres Erzgebirge, Alte Marienberger, Str. 52, 09405 Zschopau, Germany; 2 Department of Trauma, Hand and Reconstructive Surgery, Universitätsklinikum Jena, Erlanger Allee 101, 07747 Jena, Germany; 3 Department of Biochemistry, Rush Medical College, 1735 W. Harrison St, Chicago, IL 60612 USA; 4 G.E.R.N. Tissue Replacement, Regeneration & Neogenesis, Department of Orthopedics and Trauma Surgery, Medical Center - Albert-Ludwigs-University of Freiburg, Faculty of Medicine, Albert-Ludwigs-University of Freiburg, Hugstetter Straße 55, 79106 Freiburg, Germany; 5 Massachusetts Institute of Technology, Center for Biomedical Engineering, 500 Technology Sq, Cambridge, MA 02139 USA

**Keywords:** Chondrocyte, Articular cartilage, De-differentiation, Re-differentiation, Monolayer expansion, Alginate bead culture

## Abstract

**Background:**

Autologous chondrocyte implantation (ACI) is a therapy for articular cartilage and osteochondral lesions that relies on notch- or trochlea-derived primary chondrocytes. An alternative cell source for ACI could be osteochondritis dissecans (OCD) fragment-derived chondrocytes. Assessing the potential of these cells, we investigated their characteristics ex vivo and after monolayer expansion, as monolayer expansion is an integral step of ACI. However, as monolayer expansion can induce de-differentiation, we asked whether monolayer-induced de-differentiation can be reverted through successive alginate bead culture.

**Methods:**

Chondrocytes were isolated from the OCD fragments of 15 patient knees with ICRS grades 3–4 lesions for ex vivo analyses, primary alginate bead culture, monolayer expansion, and alginate bead culture following monolayer expansion for attempting re-differentiation. We determined yield, viability, and the mRNA expression of aggrecan and type I, II, and X collagen.

**Results:**

OCD fragment-derived chondrocyte isolation yielded high numbers of viable cells with a low type I:II collagen expression ratio (< 1) and a relatively high aggrecan and type II and X collagen mRNA expression, indicating chondrogenic and hypertrophic characteristics. As expected, monolayer expansion induced de-differentiation. Alginate bead culture of monolayer-expanded cells significantly improved the expression profile of all genes investigated, being most successful in decreasing the hypertrophy marker type X collagen to 1.5% of its ex vivo value. However, the chondrogenic phenotype was not fully restored, as the collagen type I:II expression ratio decreased significantly but remained > 1.

**Conclusion:**

OCD fragment derived human chondrocytes may hold not yet utilized clinical potential for cartilage repair.

**Electronic supplementary material:**

The online version of this article (10.1186/s12891-018-2079-6) contains supplementary material, which is available to authorized users.

## Background

Articular cartilage (AC) provides a low-friction interface for joint movement and distributes the forces that occur within the musculoskeletal system to the underlying subchondral bone. AC lesions are a common clinical problem because they do not heal spontaneously and often progress to higher grade AC lesions and, over time, to osteoarthritis (OA) [1]. Consequently, higher grade AC lesions are the target of many clinical therapies and basic science studies that aim to restore the AC layer with tissue engineered implants or induced hyaline-like AC. The use of autologous chondrocytes from non-weight bearing regions of AC that are seeded into a scaffold for implantation is one such method, termed autologous chondrocyte implantation (ACI). ACI is a clinically successful therapy for AC [2] and osteochondral lesions [3, 4]. However, as large-scale, degenerative AC and osteochondral lesions are a major focus of our field [5], the improvement of currently used ACI techniques and materials and the assessment of novel cell sources will gain more importance.

A crucial step in generating implants for ACI is the expansion of primary chondrocytes in monolayer culture to increase the number of available chondrocytes that are seeded into an implantable scaffold. Chondrocyte de-differentiation in extended monolayer expansion [6] limits the time of monolayer expansion, and, thus, the amount of chondrocytes that can be generated for ACI. In this context, alternative cell sources that could be used instead or in combination with autologous chondrocytes from classically used biopsy sites such as the intercondylar notch are highly interesting. Other studies focused on lesion chondrocytes [7], OA-chondrocytes [8–10], and on progenitor cells [11], as these can be differentiated in vitro into desired lineages [12–14]. Potentially, an attractive source for autologous chondrocytes for knee ACI could be the osteochondral fragment that dislocates from an osteochondritis dissecans (OCD) lesion, as an older study reported that a large OCD defect of the weight-bearing knee joint surface was treated by transplantation of an autogeneic osteochondral fragment [15]. In this context, we have previously demonstrated that human chondrocytes isolated from OCD fragments are viable [16] and, compared to notch chondrocytes from the same human joints, have an comparable mRNA expression of AC tissue engineering-relevant types I and II collagen [17]. Also, the ratio of type I to II collagen, which presents the balance between a chondrogenic vs. a de-differentiated phenotype, was comparable between OCD fragment and notch chondrocytes, indicating a chondrogenic phenotype in both OCD fragment and notch chondrocytes [17]. However, monolayer expansion, which represents an important step for producing the cell numbers needed for generating ACI implants, changed the mRNA expression profiles of clinically used notch chondrocytes but also of OCD fragment chondrocytes towards a de-differentiated phenotype [17].

In the present study, we asked the question whether human OCD fragment-derived chondrocytes, after they were intentionally de-differentiated through monolayer-expansion, can be re-differentiated using the alginate bead system [18] as a three-dimensional (3D) ACI model. Thus, we investigated ex vivo chondrocyte yield, viability, morphology, and the aggrecan and types I, II, and X collagen mRNA expression profiles of human OCD fragment-derived chondrocytes. We compared these ex vivo characteristics with those after monolayer expansion, after primary alginate bead culture, and after alginate bead culture following monolayer expansion. Collectively, such information is relevant for using human OCD fragment-derived chondrocytes for clinical ACI but, unfortunately, this information has been unavailable until now.

## Methods

### Articular cartilage

**Fig. 1:**
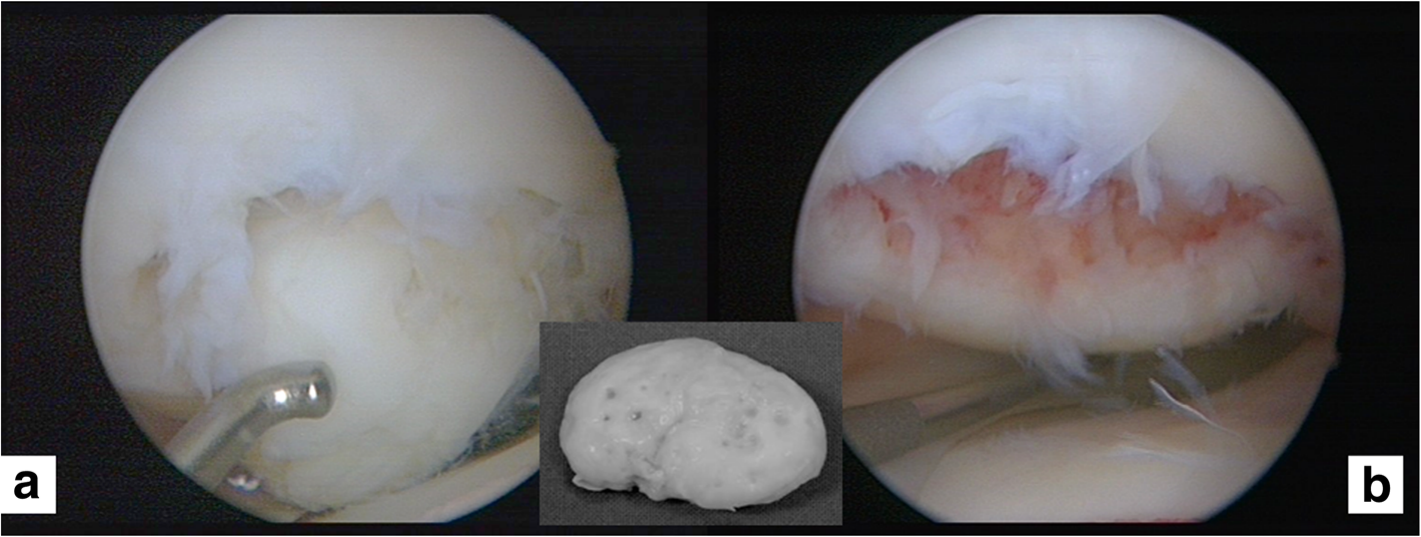
Arthroscopic images of an osteochondritis dissecans (OCD) lesion ICRS grade 4 of the medial femoral condyle in a 24 year old male patient*.* **a** Gives the initial finding at arthroscopy. **b** Illustrates the lesion after debridement and the insert shows a representative image of an dislocated and arthroscopically removed OCD fragment

AC grade 4 according to the International Cartilage Repair Society (ICRS) classification system [19] from the dissected OCD fragments of the knee joints of 15 OCD patients was used (Fig. 1). The associated demographic data of these patients are shown in Table 1. Routine MRI was used to confirm OCD diagnosis and general recommendations on inclusion and exclusion criteria were respected [2, 20]. During the initial knee arthroscopy for generating AC biopsies for ACI, OCD-fragments were harvested (Fig. 1) and transferred in standard medium to our research laboratory for further analyses. As routine surgical therapy, the OCD-affected bone was restored if necessary and matrix-associated ACI was used as previously described [21] using Novocart® 3D (TETEC, Reutlingen, Germany).

**Table 1:** Details of the specimens used in this study

Number of knees	15
Mean age (years) (range)	28 (16–49)
Male: female	10: 5
Site of OCD	11 medial, 4 lateral
Mean defect size (cm^2^) (range)	4.6 (3–8)

### Cell isolation and culture

Chondrocytes were isolated from the biopsy material designated to this study by sequential protease digestion for 1 h in 0.2% pronase, followed by overnight digestion in 0.025% collagenase-P in DMEM / F-12 medium (GIBCO BRL; Life Technologies, Grand Island, New York), supplemented with 5% autologous serum and 50 μg gentamicin / ml. The released cells were counted manually and cell viability was assayed by Trypan blue exclusion. The chondrocytes were washed three times in phosphate-buffered saline (PBS). An aliquot of 10,000 cells was dissolved in RNA extraction buffer (TRIzol Reagent; Life Technologies, Gaithersburg, Maryland) for molecular analysis (analysis 1 ex vivo). Other isolated chondrocytes were cultured in alginate beads (see below) for 3 weeks, followed by successive analysis (analysis 2 alginate). The remaining isolated chondrocytes that were not used for alginate culture were seeded in low-density monolayer culture for 3 weeks, comparable to the chondrocyte expansion routinely performed for ACI. This corresponded to approximately 2–3 passages. The chondrocytes in alginate and monolayer were cultured at 37 °C and 5% CO2 in standard DMEM / F12 feeding medium supplemented with 5% autologous serum and 50 μg gentamicin. The medium was changed every other day. At the time of confluence the monolayer chondrocytes were liberated by trypsin digestion and washed three times in PBS. An aliquot of 10,000 chondrocytes was dissolved in RNA extraction buffer (TRIzol Reagent) for molecular analysis (analysis 3 monolayer). The remaining monolayer-expanded chondrocytes were cultured in alginate beads (see below) for another 3 weeks followed by subsequent analyses (analysis 4 monolayer & alginate).

### Preparation and culture of alginate beads

The isolated chondrocytes were encapsulated in alginate beads at a density of 4 × 10^6^ cells / ml of alginate gel, as described by [22] modified by [18]. Briefly, the cells were suspended in sterile 0.15 M NaCl containing low-viscosity alginate gel (1.2%), then slowly pressed through a 22 gauge needle in a dropwise fashion into a 102 mM CaCl_2_ solution. After instantaneous gelation the beads were allowed to polymerize further for a period of 10 min in the CaCl_2_ solution. After 1 wash in 10 volumes of 0.15 M NaCl and 3 washes in 10 volumes of Ham’s F12 / DMEM medium, the beads were finally placed in standard culture medium (Ham’s F-12 / DMEM medium (50 / 50) with 10% FBS, 50 mg / ml gentamicin and 25 mg / ml ascorbic acid). Each bead contained an average chondrocyte number of 44 ± 2 × 10^3^ cells / bead. Nine beads were cultured per well of a 24-well plate. The cells were incubated at all stages in a humidified atmosphere of 5% CO_2_ at 37 °C and maintained by medium change 3 times per week with 1.5 ml medium/well. After 3 weeks, the beads were dissolved with 1 ml of 55 mM sodium citrate, 0.15 M NaCl, pH 6.05, at 25 °C for 20 min and the chondrocytes were recovered by centrifugation [18].

### Quantitative real-time polymerase chain reaction (qRT-PCR)

Chondrocytes were dissolved in RNA extraction buffer (1 ml TRIzol Reagent), and Chloroform (200 μl) was added. After shaking and incubation for 3 min at room temperature, the samples were centrifuged for 15 min at 12000 g (4 °C) for phase separation. Total RNA was then precipitated from the aqueous phase with isopropyl alcohol (0.5 ml, 10 min, room temperature), and centrifuged for 10 min at 12000 g and 4 °C. Subsequently, the RNA was washed twice with 700 μl of 75% ethanol, dried at 42 °C, and re-dissolved in nuclease-free water, followed by digestion of genomic DNA using RNase free DNase (Qiagen, Hilden, Germany). Total RNA yield was assessed by spectrophotometry at 260 nm. The cDNA was synthesized from 1 μg of total RNA using the Omniscript RT Kit (Qiagen, Hilden, Germany) according to the manufacturer’s protocol. Quantitative real-time PCR was performed in an iCycler iQ (Bio-Rad Laboratories, Hercules, California) in 20 μl reaction volume containing 9.4 μl cDNA, 0.3 μl forward primer, 0.3 μl reverse primer and 10 μl iQ SYBR Green supermix (Bio-Rad Laboratories). All investigated target genes and the sequences of their corresponding primers are listed in Table 2. The primers were validated by gel analysis, are listed in the NCBI reference sequence database and have been used in our previous studies [16, 17, 23]. The sequence specificity was confirmed by BLAST searches. The reactions were run with appropriate controls (no template) to assess cross-contamination. The cycling parameters were: 95 °C for 3 min; 40 cycles: 94 °C for 20 s, annealing at 60 °C for 20 s, extension at 72 °C for 20 s; and 95 °C for 1 min. The PCR was evaluated by melting curve analysis. Data were calculated by the cycle threshold method (expressed as 2^-ΔCt^), normalized to glyceraldehyde phosphate dehydrogenase (GAPDH) mRNA expression, and are presented as ratios. Amplification efficiencies and GAPDH stable expression as reference gene were confirmed for our previous studies [16, 17, 23], in which the same protocol was used.

**Table 2:** Primers used for the polymerase chain reaction (PCR); F: forward primer sequence, R: reverse primer sequence

Gene Name	PubMed Accession Number	Sequence
GAPDH	BT006893	F: CAT CAC TGC CAC CCA GAA GA
		R: CCT GCT TCA CCA CCT TCT TG
Type X collagen	X98568	F: CCT CTT GTT AGT GCC AAC CAG
		R: GAG CCA CTA GGA ATC CTG AG
Type I collagen (COL1A2)	XM_029245	F: CTC TGC GAC ACA AGG AGT CT
		R: ATC TTC ACC AGC CTT GCC AG
Type II collagen (COL2A1)	X16711	F: CAA CAC TGC CAA CGT CCA GAT
		R: CTG CTT CGT CCA GAT AGG CAA T
Aggrecan	NM_013227	F: ACT TCC GCT GGT CAG ATG GA
		R: TCT CGT GCC AGA TCA TCA CC

### Histology

A representative AC sample was fixed in 4% paraformaldehyde and embedded in paraffin. Paraffin sections of 5 μm thickness were prepared. Conventional staining included a routine dual stain with hematoxylin / eosin for cellular distribution and safranin-O / fast green for proteoglycan content. Alkaline phosphatase detection was performed using a commercially available kit according to the manufacturer’s protocol (Pierce). Immunohistochemistry was performed with a mouse monoclonal antibody against human type I collagen (Oncogene Research Products, Boston, Massachusetts) and a rabbit polyclonal antibody against human type II collagen (Abcam, Cambridge, UK) and type X collagen (kindly provided by Klaus von der Mark, University of Erlangen, Germany), as described earlier in our and other studies [16, 24].

### Statistical analyses

Analyses were performed using SigmaPlot 11.0 and SigmaStat 3.0 (SPSS Inc., Chicago, USA). Analysis of variance (ANOVA) and normality testing were performed for all groups. Differences between the groups before or after cell culture were assessed with the Kruskal–Wallis one-way ANOVA on ranks. If the ANOVA tests for comparing chondrocytes before vs. after cell culture were statistically significant, an all-pairwise multiple comparison procedure (Student-Newman-Keuls Test) was performed to isolate the group or groups that differed from the others. Correlations were tested for significance by the Spearman’s rank order correlation test. A *p* < 0.05 was considered statistically significant.

## Results

### Histology and immunohistochemistry

**Fig. 2:**
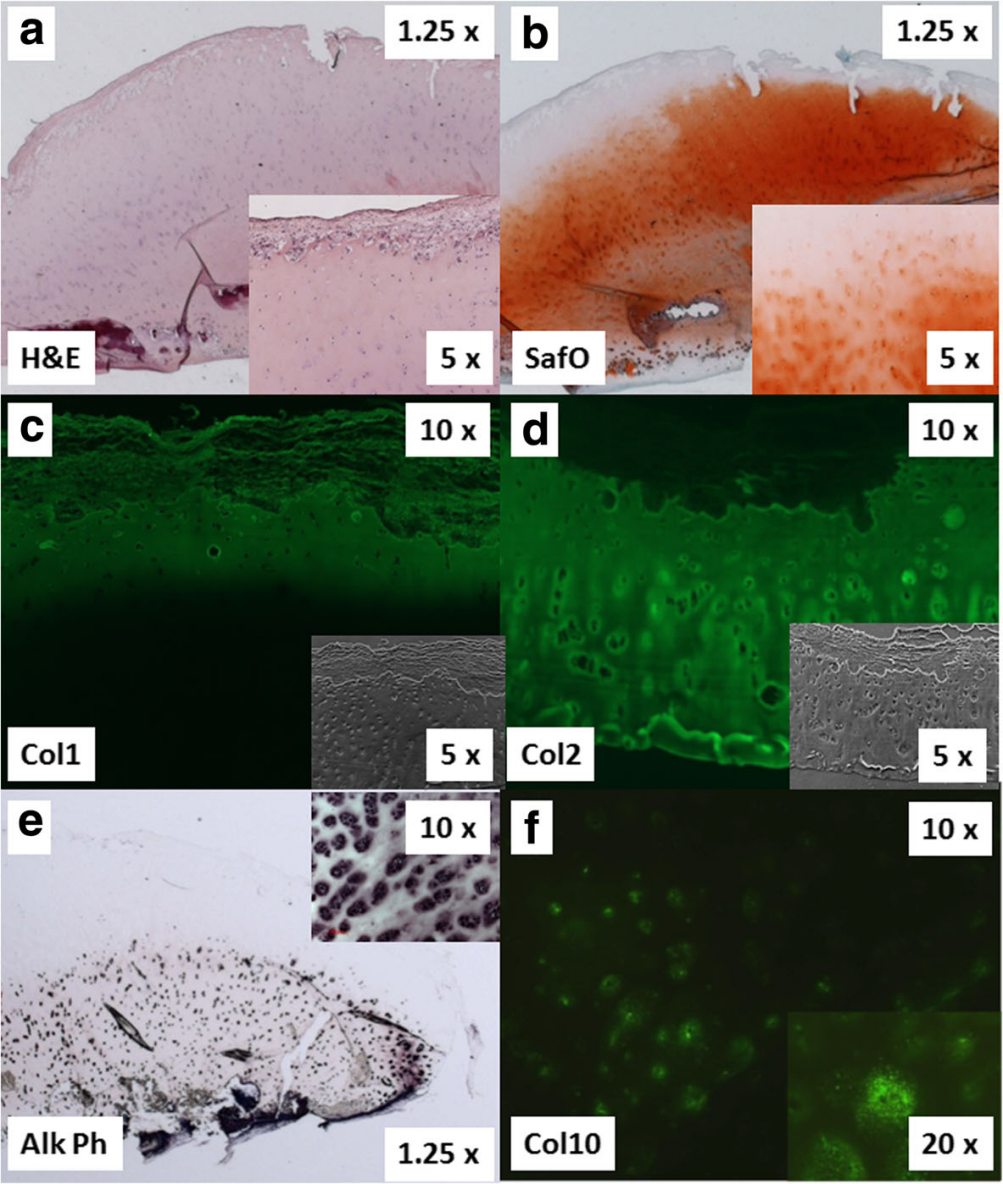
Histology of a representative OCD fragment from the debrided ICRS grade 4 lesion of the medial condyle of a 22 year old patient. **a** Depicts a hematoxylin / eosin stain and (**b**) a safranin-O / fast green stain. **c**, **d** Shows immunostaining for types I (**c**) and II collagen (**d**) together with phase contrast images as inserts. **e** Shows alkaline phosphatase staining. **f** Shows immunostaining for type X collagen. Original magnifications of 1.25 x (**a**, **b**), 10 x (**c**-**f**) and insert magnifications of 5 x (**a**-**d**), 10 x (**e**, **f**)

The histological characteristics of a representative OCD AC fragment derived from the medial femoral condyle are shown in Fig. 2. Two overviews at 5× magnification depict the irregular superficial zone stained with hematoxylin and eosin and with safranin-O and fast green (Fig. 2a,b), illustrating a homogenous chondrocyte distribution throughout the extracellular matrix. The safranin-O and fast green stain (Fig. 2b) revealed that the central part of the fragment contained proteoglycans, whereas the superficial zone was depleted. Interestingly, there was an increased pericellular staining in the deep zone, indicating in increased proteoglycan deposition I in that zone (insert in B). Note the presence of a fibrous-appearing layer covering the superficial zone (Fig. 2a insert), whose immunostaining for type I and II collagen is depicted in Fig. 2c,d, revealing that type I collagen was present within the superficial zone and the fibrous layer and that collagen type II was present throughout the tissue. The phase contrast pictures (inserts in C and D) depict the entire section. Interestingly, alkaline phosphatase was detected throughout the tissue (Fig. 2e) in the pericellular area (insert in E), which was also true for type X collagen (Fig. 2f). Collectively, these images demonstrated that the examined OCD fragment contained the types I, II, and X collagen.

### Chondrocyte yield, viability, and morphology

Approximately 0.73 ± 0.33 × 10^6^ chondrocytes per gram wet weight were extracted. The mean cell viability was in all groups above 88% and no significant differences between the groups were noted. After isolation the suspended chondrocytes depicted a round phenotype but once seeded onto a tissue culture plate in low density the morphology had changed as expected to a more irregular and fibroblastic-appearing phenotype. After 3 weeks of monolayer expansion, the chondrocytes had regained this phenotype. During subsequent cultivation in alginate beads for another 3 weeks, the chondrocytes had changed to a round morphology. The number of isolated cells per OCD fragment and the viability of the individual cell cultures that were derived from these fragments is given in Table 3.

**Table 3:** Number of isolated cells per OCD fragment and viability of the individual cell cultures that were derived from these fragments

OCD Fragment	Cell number (× 100.000/g wet weight)	Viability after Cell Culture (% of total)			
		Analysis 1	Analysis 2	Analysis 3	Analysis 4
1	0.72	91	89	93	92
2	0.36	86	88	87	89
3	0.8	75	79	81	85
4	0.56	96	95	94	91
5	1.24	90	96	90	93
6	0.42	89	85	93	89
7	1.01	93	93	85	89
8	0.77	82	91	87	85
9	0.9	87	85	89	96
10	0.61	93	87	94	95
11	1.23	95	94	92	93
12	0.31	96	97	95	91
13	0.37	87	92	89	84
14	0.41	91	93	92	90
15	1.22	92	93	90	92

### Molecular analyses

**Fig. 3:**
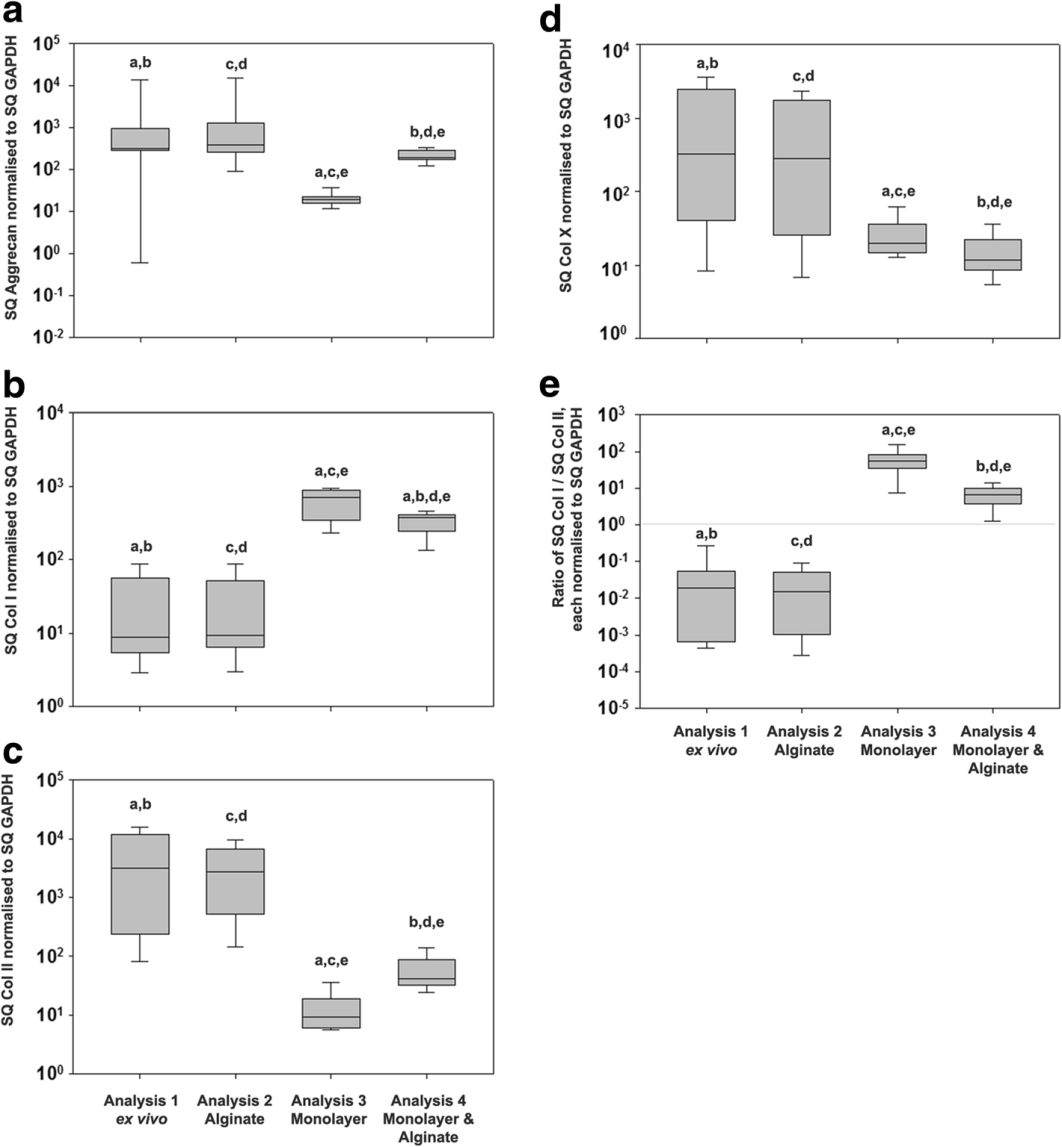
Comparison of OCD fragment-derived chondrocyte mRNA expression ex vivo, after alginate bead culture, after monolayer culture, and after monolayer culture followed by successive alginate bead culture. This figure depicts the mRNA expression of aggrecan (**a**), types I (**b**), II (**c**), and X collagen (**d**) of OCD fragment-derived chondrocytes. (**e**) gives the ratio of type I to II collagen mRNA expression, which illustrates the balance between a functional chondrocyte phenotype (value < 1) as in intact cartilage and a de-differentiated in vitro phenotype (value > 1). The transition between both phenotypes is indicated by a grey line. Data are shown as box plots with each box representing the 25th and 75th percentile and the whiskers the 10th and 90th percentiles. Solid lines within the box indicate the median. Significant differences of pairwise comparisons are indicated by small letters. E.g. “a” indicates that the comparison of the type I collagen mRNA expression in (**b**) between analysis 1 (ex vivo) and analysis 3 (monolayer) was significant

Molecular analyses revealed patient-specific mRNA expression variation on the order of magnitudes. Aggrecan mRNA expression (Fig. 3A) was comparable immediately after cell isolation (analysis 1) and after primary culture in alginate beads (analysis 2), which increased the expression 1.08-fold but without any significant difference between the two groups. After expansion in monolayer culture (analysis 3), aggrecan mRNA expression was decreased to 0.007-fold, compared to cell isolation and to alginate bead culture, and the difference was significant (*p* < 0.001), compared to both groups. After alginate bead culture following monolayer expansion (analysis 4), aggrecan mRNA expression was significantly increased 10.2-fold (*p* < 0.001), compared to monolayer expansion only (analysis 3). However, the expression levels were significantly lower than after isolation (0.07-fold, *p* < 0.001) or 3D culture I (0.07-fold, *p* < 0.001). Additional information on mRNA expression and cell viavility can be found in the supplementary file (Additional file 1).

Type I collagen mRNA expression (Fig. 3B) was increased after monolayer culture expansion (analysis 3), compared to ex vivo (analysis 1, 24.1-fold) and to alginate bead culture (analysis 2, 24.8-fold), and the difference was significant (*p* < 0.001), compared to both groups. After alginate bead culture following monolayer expansion (analysis 4), type I collagen mRNA expression was significantly decreased to 0.5-fold (*p* < 0.001), compared to monolayer expansion only (analysis 3). However, the expression levels were significantly higher than ex vivo (analysis 1, 12.5-fold, *p* < 0.001) or after alginate bead culture (analysis 2, 12.9-fold, *p* < 0.001).

Type II collagen mRNA expression (Fig. 3C) was not significantly different after primary culture in alginate beads (analysis 2) vs. ex vivo (analysis 1). After expansion in monolayer culture (analysis 3), type II collagen mRNA expression was decreased to 0.003-fold, compared to ex vivo (analysis 1) and 0.004-fold compared to alginate bead culture (analysis 2). The difference was significant (*p* < 0.001), compared to both groups. After alginate bead culture following monolayer expansion (analysis 4), type II collagen mRNA expression was significantly increased to 4.5-fold (*p* < 0.001), compared to monolayer expansion (analysis 3). However, the expression levels were significantly lower than ex vivo (analysis 1, 0.012-fold, *p* < 0.001) or alginate bead culture (analysis 2, 0.019-fold, *p* < 0.001).

Type X collagen mRNA expression (Fig. 3D) was not significantly changed after alginate bead culture (analysis 2), compared to ex vivo (analysis 1). After monolayer expansion (analysis 3), the type collagen X mRNA expression was significantly decreased to 0.03-fold, compared to ex vivo (analysis 1, *p* < 0.001). It further decreased after alginate bead culture following monolayer expansion (analysis 4, *p* < 0.001) by 0.6-fold, compared to monolayer expansion (analysis 3), or decreased 0.01-fold, compared to ex vivo (analysis 1).

The ratio of type I to II collagen mRNA expression (Fig. 3E), which we calculated for each OCD fragment from the types I and II collagen mRNA expression values, was comparable ex vivo (analysis 1, 0.026 ± 0.035) and after alginate bead culture (analysis 2, 0.054 ± 0.09, 3D culture I), as the trend towards a 0.49-fold decrease after alginate bead culture did not reach significance. For both groups the ratio values were < 1, indicating a higher type II than I collagen mRNA expression and, thus, a healthy chondrocyte function. After monolayer expansion (analysis 3), the ratio was increased to 69.61 ± 47.7 and the increase was significant, compared to both groups (*p* < 0.001). After alginate bead culture following monolayer expansion (analysis 4), the ratio value was 7.23 ± 4.2, which indicated a significant recovery of the ratio, compared to monolayer expansion (analysis 3, *p* < 001, Fig. 3).

We also analyzed the data to uncover potential differences between medial and lateral defect locations. However, the mRNA expression data obtained from analyses 1 to 4 for chondrocytes from the medial vs. lateral defect locations were not significantly different (except type I collagen expression in the group analysis 2).

### Correlation analyses

Ex vivo aggrecan mRNA expression levels (analysis 1) correlated significantly and positively with those after culture in alginate beads (analysis 2, *p* < 0.001, correlation coefficient cc: 0.988). Moreover, the ex vivo mRNA expression of collagen type I (analysis 1) correlated significantly and positively with the expression levels in primary alginate bead culture (analyses 2, *p* = 0.005, cc: 0.682). The same was true for the collagen types II (*p* < 0.001, cc: 0.989) and X (*p* < 0.001, cc: 0.986) expression levels, indicating that a high ex vivo expression of aggrecan, collagen types I, II, and X were associated with a high expression in primary alginate culture.

Interestingly, the mRNA expression levels after monolayer expansion (analysis 3) of all genes of interest correlated significantly and positively with the expression levels after alginate bead culture following monolayer expansion (analysis 4; aggrecan: *p* < 0.001, cc: 0.913; collagen type I: *p* < 0.001, cc: 0.973; collagen type II: *p* < 0.001, cc: 0.987; collagen type x: *p* < 0.001, cc: 0.981). This indicated that OCD fragment-derived chondrocytes with a relatively high mRNA expression of these genes in monolayer culture had also high expression levels in subsequent alginate bead culture following monolayer expansion. Together, these data indicated that patient-specific mRNA expression level variations were maintained across the culture systems used.

## Discussion

The present study asked whether intentionally in monolayer de-differentiated human OCD fragment-derived chondrocytes can be re-differentiated by using the alginate bead system [18] as an in vitro 3D ACI model. First, we demonstrated that chondrocytes can be isolated from the dissected OCD fragments of human patients with a sufficient yield and viability that was consistent with the literature [16]. Furthermore, ex vivo OCD fragment-derived chondrocytes displayed a functional chondrocyte phenotype, which was determined by calculating the ratio of type I to II collagen mRNA expression. The ratio was comparable to those of chondrocytes from the notch and from AC lesions of human knee joints [7], suggesting a comparable chondrogenic phenotype of cells from these three AC locations. As expected, monolayer expansion led to chondrocyte de-differentiation [6], based on an irregular and fibroblastic-appearing phenotype and a decreased aggrecan and type II collagen mRNA expression and simultaneous increases in the type I collagen mRNA expression and the type I to II collagen expression ratio, compared to ex vivo. Subsequent alginate bead culture of the monolayer-expanded, de-differentiated OCD fragment-derived chondrocytes led to changes in cell morphology and mRNA expression that were consistent with re-differentiating the de-differentiated OCD fragment-derived chondrocytes. However, although we noted increased aggrecan and type II collagen mRNA expression levels and decreases in the type I collagen mRNA expression and the type I to II collagen expression ratio, the resulting mRNA expression values were significantly different from those measured ex vivo. Thus, induced re-differentiation was only partially successful in recovering the mRNA expression profiles. Interestingly, the type X collagen mRNA expression of OCD fragment-derived chondrocytes was lowest after re-differentiation of monolayer-induced de-differentiation in alginate beads, compared to primary alginate bead culture, monolayer expansion, and even compared to ex vivo. That monolayer expansion led to a significant reduction in the type X collagen mRNA expression or, as an alternative explanation, to the selection of a less hypertrophic cell pool was consistent with our previous study [17]. Collectively, this study demonstrated that ex vivo OCD fragment-derived chondrocytes were characterized by a chondrogenic but also hypertrophic phenotype, based on the low type I to II collagen expression ratio, and at the same time the high type X collagen mRNA expression. After monolayer expansion, the re-differentiation through alginate bead culture was most effective in modulating the mRNA expression of type X collagen and, to a lesser extent, of aggrecan but was not effective in lowering the collagen type I to II expression ratio to values below 1. Thus, induced re-differentiation led to a marked reduction of the hypertrophic phenotype to approximately 1.5% of its ex vivo value but did not fully restore the chondrogenic phenotype, measured as type I to II collagen expression ratio. Collectively, OCD fragment-derived cells are interesting chondrogenic but also hypertrophic chondrocytes, whose re-differentiation with the here described method was effective in modulating hypertrophic but not chondrogenic characteristics. Subsequent procedures for improving the chondrogenic properties of OCD-fragment-derived cells would greatly enhance their clinical potential.

For inducing chondrocyte re-differentiation, previous studies have used alginate bead and pellet culture [25], alginate bead culture combined with serum or growth factor cocktails [26], hypoxic pellet culture [27], porous scaffold surfaces for the co-culture with mesenchymal stromal cells [28], agarose [29] and photocrosslinkable hydrogels [30], as well as the chimeric Activin A / BMP2 ligand AB235 [31]. These studies investigated the re-differentiation of human OA-chondrocytes obtained during joint replacement procedures [25–27, 30, 31] and of porcine and bovine chondrocytes [28, 29]. As human chondrocytes derived from dislocated OCD fragments have not been investigated, the present study added an important part to the literature on chondrocyte re-differentiation. Re-differentiation in 3D can be enhanced by adding human serum or transforming growth factor beta1 and insulin-transferrin-selenium-linoleic acid-bovine serum albumin [26] or human mesenchymal stromal cells [28], by using hypoxic conditions [27], by varying adhesion site density but not stiffness [29], and by choosing re-differentiation-supporting biomaterials such as hyaluronic acid hydrogels [30]. In the present study, the re-differentiation-associated changes in mRNA expression were comparable to [25], in which changes in mRNA expression were induced via 3D alginate bead cultures after chondrocyte de-differentiation. In line with our study, [25] reported a significantly decreased type X collagen mRNA expression and increased types I and II collagen and aggrecan expression. In this respect, the effects of alginate bead culture for re-differentiating OCD fragment-derived chondrocytes appeared comparable to human OA-chondrocytes obtained during joint replacement procedures.

A recent study investigated the frequency of OCD fragment chondrocyte cultures and reported that it was possible to culture chondrocytes from traumatic osteochondral fragments within a year of injury but not from fragments due to OCD [32]. This interesting study suggested that the time elapsed between loose body formation and fragment excision was the main factor affecting the cell culture setup. Our data does not allow drawing a direct comparison, as we did not record the time between loose body formation and cell isolation. However, we noted two methodological differences between the studies. Whereas [32] used enzymatic digestion with collagenase-A overnight, the present study used a sequential digestion for 1 h in pronase, followed by overnight digestion in collagenase-P. Another independent study [33] used the same sequential digestion used here and reported a comparably high viability in each studied OCD fragment but did not attempt cell culture. Additionally, the culture conditions between [32] and our study differed, as [32] used 10% of fetal bovine serum and the present study used 5% autologous serum. Together, these methodological differences may explain in part the difference in the number of successfully established OCD fragment-derived chondrocyte cultures. Thus, a head-to-head comparison of these different methods together with information on time since disease onset as suggested by [32] may help to determine the optimal conditions and the expected frequency of successfully established OCD fragment-derived chondrocytes.

It is worthwhile to mention that recent research on healthy (porcine) AC identified GAPDH as one of the most stably expressed genes [34], whereas it is considered a less stable reference gene in full osteoarthritis (human) patients older than 60 years [35]. As the choice of the appropriate reference gene depends on the individual experimental set-up [36], we had confirmed the suitability of the chosen reference gene for our previous studies. However, the method could be improved upon because MIQE guidelines encourage using more than one reference gene [37]. Generally, the methodology of the present study was chosen to allow better comparisons to our previous studies [16, 17, 23, 38]. A previous study comparing the here chosen method of SYBR Green based detection to TaqMan based detection found TaqMan to be more sensitive and to generate lower calculated expression levels than the SYBR Green assay, suggesting that any discussion of gene expression levels needs to be linked to the qPCR method used in the analysis [39]. However, this was not of great concern in the present study because our main conclusions were based on the *expression ratio* of type I to II collagen as well as *relative* changes in aggrecan and types II and X collagen mRNA expression. Also in a methods context, it is worth mentioning that de-differentiation of monolayer cultured chondrocytes is a progressive process that depends on passage and culture time [26, 40]. Thus, one can chose either time or passage number for culture standardization. Here, we chose a standardized culture time of 3 weeks, according to [26], which corresponded to approximately 2–3 passages. We did not analyze the re-differentiation status as a function of chondrocyte passage number, although this would be interesting data.

Although we calculated the number of isolated cells per OCD fragment wet weight, we did not record the total number of cells that were contained within the fragment at the time of isolation because we allocated the cell suspension into 4 cell culture groups. Thus, we cannot directly assess whether all OCD fragments would have qualified for ACI, based on cell numbers. However, the sparse literature on this topic documents that a range of cell densities is being used for ACI [41]. Additionally, in the personal experience of the authors OCD fragments can be processed for later ACI as a future therapy option, regardless of the initial cell number.

As the cell quality affects the clinical outcome of ACI [42], it is helpful to mention that OCD fragment-derived chondrocyte exhibited a patient-specific variation of the mRNA expression that was on the order of magnitudes. Specifically, the chondrocytes from certain donors had a more chondrogenic mRNA expression profile than the cells from other donors. These variations were not associated with any patient demographics. However, we noted through our correlation analyses that the patient-specific variations in the mRNA expression levels were maintained across the culture systems used. This indicated that patient-specific expression characteristics measured ex vivo were maintained in vitro throughout de-differentiation and induced re-differentiation. Whether such patient-specific expression characteristics can potentially be preserved after implantation is not known. In the context of cell quality, it appears also important to choose an appropriate cell source for ACI. Studies that examine joint locations are valuable, as chondrocytes from diverse locations differ in many characteristics. For example, their metabolic characteristics vary across joints [10, 43]. Moreover, the cellular distribution within the native tissue varies between individual joints [44] and even joint surfaces within the same joint [44, 45] and between different stages of OA [46]. In clinical routine, ACI utilizes biopsies of macroscopically intact cartilage from non-weight bearing areas of the (knee) joint [47] such as the intercondylar notch and the trochlea. To determine the “best cell source” for regenerative cartilage therapies, one study investigated chondrocytes isolated from AC lesions vs. from healthy AC [48]. Interestingly, chondrocytes from the AC lesion performed better than from healthy AC and it was concluded that AC lesions could be viable donor sites for ACI. In this regard, chondrocytes from alternative cell sources such as AC lesions [48] may hold clinical potential for AC repair but need further investigation, as – in the case of AC lesions – conflicting evidence has been reported [49]. However, in the case of knee OCD fragment-derived chondrocytes, the here reported biological characteristics are in accordance with our previous studies on this topic [16, 17] and clinical experience for OCD repair of the talus is promising [50]. Thus, we also analyzed the here reported data to uncover potential differences between medial and lateral defect locations. However, our data suggested that defect location did not have a significant effect on the ex vivo mRNA expression profile or the expression profiles after alginate culture, monolayer expansion, or alginate culture after monolayer expansion-induced de-differentiation.

## Conclusion

Collectively, human OCD fragment-derived chondrocytes depict chondrogenic but also hypertrophic characteristics, whose re-differentiation with the here described method was effective in modulating hypertrophic characteristics and to a lesser extent chondrogenic characteristics. The here presented data are important for better understanding and ultimately utilizing the clinical potential of human OCD fragment-derived chondrocytes for AC repair.

## Additional file

Additional file 1: mRNA expression values for collagen types I, II, and X (Col I, Col II, and Col X, respectively) and aggrecan as well as cell viability. (DOC 40 kb)
