# Supplementary material for: Human osteochondritis dissecans fragment-derived chondrocyte characteristics ex vivo, after monolayer expansion-induced de-differentiation, and after re-differentiation in alginate bead culture
Source: BMC Musculoskelet Disord. 2018 May 24;19:168. doi: 10.1186/s12891-018-2079-6 (PMC5968539; doi:10.1186/s12891-018-2079-6)
Supplement: Supplementary file 1 — mRNA expression values for collagen types I, II, and X (Col I, Col II, and Col X, respectively) and aggrecan as well as cell viability. (DOC 40 kb) [file 12891_2018_2079_MOESM1_ESM.doc]

Additional file 1: **mRNA expression values for collagen types I, II, and X (Col I, Col II, and Col X, respectively) and aggrecan as well as cell viability.**

Values are given *ex vivo* after isolation (analysis 1), after primary alginate culture (analysis 2), after monolayer expansion (analysis 3), and after alginate bead culture following monolayer expansion (analysis 4). Given are the mean ± standard deviation (upper values) and the median (middle value) and range (lower values).

|  | **Aggrecan** | **Col I** | Col II | **Col X** | **Col I/II** | **Cell**  **Viability**  **(%)** |
| --- | --- | --- | --- | --- | --- | --- |
| **Analysis 1:**  After cell liberation | 2997 ± 5605  327  (0.15–15782) | 26.3 ± 31.7  8.75  (.46–92.2) | 5226 ± 6270  3140  (73–16104) | 1064 ± 1383  326  (5.94-4171) | 0.054 ± 0.09  0.019  (0.001-0.278) | 89.5 ± 5.62  91  (75-96) |
| **Analysis 2:**  After alginate bead culture | 3225 ± 5884  390  (76.3-14884) | 25.5 ± 32.3  9.25  (1.58-106) | 3431 ± 3600  2746  (130-10956) | 712 ± 947  278  (4.56-2952) | 0.026 ± 0.035  0.015  (0.001-0.124) | 90.5 ± 4.93  92  (79-97) |
| **Analysis 3:**  After monolayer expansion | 21.5 ± 8.43  19.1  (10.9-39.5) | 633 ± 255  705  (163–982) | 14.1 ± 10.6  9.31  (5.42–36.2) | 27.1 ± 17.8  19.5  (12.6–65.1) | 69.6 ± 47.7  56.2  (4.51–157) | 90 ± 3.86  90  (81–95) |
| **Analysis 4:**  After alginate bead culture following monolayer expansion | 220 ± 77.5  199  (121-410) | 329 ± 117  373  (89.5-513) | 64 ± 41.8  41.3  (24.5-143) | 15.7 ± 10.9  11.8  (5.45-42.6) | 7.23 ± 4.22  6.79  (0.65-14.9) | 90.3 ± 3.56  91  (84-96) |
